# NeoCLIP: a self-supervised foundation model for the interpretation of neonatal radiographs

**DOI:** 10.1038/s41746-025-01922-6

**Published:** 2025-09-24

**Authors:** Yixuan Huang, Puneet Sharma, Anil Palepu, Nathaniel Greenbaum, Andrew Beam, Kristyn Beam

**Affiliations:** 1 Department of Biostatistics, Harvard T.H. Chan School of Public Health, Boston, MA USA; 2 Division of Neonatology, Emory University School of Medicine, Atlanta, GA USA; 3 Department of Health Sciences & Technology, Massachusetts Institute of Technology, Cambridge, MA USA; 4 Department of Anesthesia, Critical Care, and Pain Medicine, Beth Israel Deaconess Medical Center, Boston, MA USA; 5 Department of Epidemiology, Harvard T.H. Chan School of Public Health, Boston, MA USA; 6 Department of Neonatology, Beth Israel Deaconess Medical Center, Boston, MA USA

**Keywords:** Paediatric research, Image processing

## Abstract

Deep learning has proven to be an excellent tool for interpreting medical images in adults and children. However, it is still underdeveloped for neonates. This study developed NeoCLIP, a novel deep contrastive learning model designed to detect pathologies and medical devices on neonatal radiographs. We retrospectively studied 4629 infants admitted to a neonatal intensive care unit in Boston, MA (2008–2023), compiling 20,154 radiographs and 15,795 corresponding reports. The cohort was randomized into training (80%), validation (10%), and test (10%) sets. NeoCLIP was trained to identify 15 radiological features and 5 medical devices relevant to neonatal intensive care. NeoCLIP achieved higher AUROC compared to controls in all labels except portal venous gas. Incorporating demographic data improved model performance, though not statistically significantly. NeoCLIP is the first deep learning model tailored for the interpretation of neonatal radiographs, surpassing the efficacy of comparable adult models.

## Introduction

Medicine has undergone a remarkable transformation in recent decades. A discipline that once relied on paper charts and radiographs has taken a dramatic shift to a more digital format. With the widespread adoption of electronic medical records and digitization of medical imaging, healthcare now generates thirty percent of global data^1^. This data has the potential to revolutionize patient care, but the volume has exceeded the capacity of human processing. However, timely progress in artificial intelligence (AI), specifically deep learning, has made it possible to interpret this data to enhance clinical care^2^.

In radiology, deep learning methodologies have been used to interpret medical imaging to make important diagnostic and management recommendations^3–5^. Vision-language contrastive learning models, in particular, have shown promise^6–10^. These models adapt general-purpose foundation models to align medical images with their corresponding reports in a self-supervised manner, allowing for the accurate identification of clinical findings on imaging without explicit training.

Despite success in adult and pediatric populations, this approach has not been systematically applied to neonates. This disparity is particularly interesting given that many common pathologies of neonatal-perinatal medicine, such as bronchopulmonary dysplasia (BPD), respiratory distress syndrome (RDS), and necrotizing enterocolitis, are either screened for or diagnosed on plain film radiographs^11–13^. These unique neonatal pathologies are of course in addition to other important indications for radiographs in critical care such as post-procedural imaging. A deep learning model that could interpret neonatal imaging could therefore improve the care of these complex pathophysiologies by expediting diagnosis and identifying subtle findings otherwise not appreciable to the human eye^14^. As such, the objective of our study was to develop a foundation model for neonatal imaging capable of interpreting radiographs to identify findings associated with common pathologies in neonatal intensive care.

## Results

We identified 7986 infants who were admitted to the NICU at BIDMC during our enrollment period. We then excluded 3357 infants who did not have a radiograph obtained during their admission, resulting in a final cohort of 4629 infants. For this cohort, we collected 20,154 radiographs with 15,795 corresponding reports. The breakdown of radiograph type in our cohort was: 10,793 chest x-rays, 3413 abdominal x-rays, and 5948 babygrams (chest and abdomen in one film). The discrepancy between the number of radiographs and reports is due to single reports that describe multiple radiographs taken in succession. We then randomized the cohort into the three sets which were distributed as follows:

1. Training set: 3731 patients (80%) with 16,204 images and 12,727 reports.
2. Validation set: 419 patients (10%) with 1915 images and 1454 reports.
3. Test set: 479 (10%) with 2035 images and 1614 reports.

The mean gestational age and weight of all three sets were similar after randomization – approximately 34 weeks and 2.4 kg respectively. All other relevant demographic data and summary of images collected are summarized in Table 1.

**Table 1:** Randomization of cohort

	Training cohort			Validation cohort			Test cohort		
	Patients (*n* = 3731)	Radiographs (*n* = 16,204)	Reports (*n* = 12,727)	Patients (*n* = 419)	Radiographs (*n* = 1915)	Reports (*n* = 1454)	Patients (*n* = 479)	Radiographs (*n* = 2035)	Reports (*n* = 1614)
**Sex (%)**									
Female	1581 (42)	7261 (45)	5716 (45)	241 (58)	769 (40)	611 (42)	265 (55)	886 (44)	702 (43)
Male	2150 (58)	8943 (55)	7011 (55)	178 (42)	1146 (60)	843 (58)	214 (45)	1149 (56)	912 (57)
**Race and Ethnicity of Mother (%)**									
Asian	403 (11)	1565 (10)	1258 (10)	43 (10)	246 (13)	183 (13)	37 (8)	230 (11)	185 (11)
Black or African American	522 (14)	2832 (17)	2202 (17)	67 (16)	412 (22)	318 (22)	63 (13)	334 (16)	269 (17)
Hispanic	649 (17)	2872 (18)	2213 (17)	77 (18)	428 (22)	320 (22)	84 (18)	421 (21)	317 (20)
White	2143 (57)	8900 (54)	7024 (55)	232 (56)	829 (43)	633 (44)	294 (60)	1049 (51)	842 (52)
Other	14 (1)	35 (1)	30 (1)	0 (0)	0 (0)	0 (0)	1 (1)	1 (1)	1 (1)
**Birth weight (g), mean (SD)**	2405 (1004)			2379 (1005)			2408 (1004)		
**Gestational age (weeks), mean (SD)**	34.4 (4.3)			34.4 (4.3)			34.5 (4.2)		
**Positive cases (%)**									
Atelectasis		5504 (34)	4459 (35)		662 (35)	514 (35)		740 (36)	614 (38)
Intestinal Atresia		138 (1)	125 (1)		27 (1)	19 (1)		25 (1)	21 (1)
Bronchopulmonary Dysplasia		1759 (11)	1528 (12)		170 (9)	144 (10)		323 (16)	288 (18)
Cardiomegaly		971 (6)	781 (6)		125 (7)	95 (7)		104 (5)	82 (5)
Endotracheal Tube Placement		6764 (42)	5255 (41)		739 (39)	564 (39)		911 (45)	729 (45)
Meconium Aspiration Syndrome		377 (2)	299 (2)		46 (2)	36 (2)		31 (2)	27 (2)
Nasogastric Tube Placement		7926 (49)	6419 (50)		970 (51)	779 (54)		987 (49)	826 (51)
Intestinal Obstruction		368 (2)	315 (2)		71 (4)	51 (4)		74 (3)	55 (3)
Pulmonary Edema		709 (4)	611 (5)		59 (3)	48 (3)		62 (3)	48 (3)
Peripherally Inserted Central Catheter Placement		2278 (14)	1597 (13)		281 (15)	193 (13)		283 (14)	218 (14)
Pleural Effusion		751 (5)	636 (5)		93 (5)	74 (5)		79 (4)	61 (4)
Pneumonia		236 (1)	208 (2)		27 (1)	24 (2)		28 (1)	25 (2)
Pneumatosis Intestinalis		489 (3)	391 (3)		54 (3)	41 (3)		53 (3)	43 (3)
Pneumothorax		924 (6)	726 (6)		71 (4)	59 (4)		72 (4)	60 (4)
Pneumoperitoneum		95 (1)	76 (1)		21 (1)	13 (1)		13 (1)	12 (1)
Portal Venous Gas		208 (1)	136 (1)		11 (1)	7 (0)		16 (1)	10 (1)
Respiratory Distress Syndrome		6154 (38)	4661 (37)		672 (35)	510 (35)		808 (40)	605 (37)
Transient Tachypnea of the Newborn		456 (3)	402 (3)		57 (3)	53 (4)		63 (3)	54 (3)
Umbilical Artery Catheter Placement		1717 (11)	1150 (9)		226 (12)			209 (10)	151 (9)
Umbilical Vein Catheter Placement		5883 (36)	3902 (31)		697 (36)			731 (36)	478 (30)

**Fig. 1:**
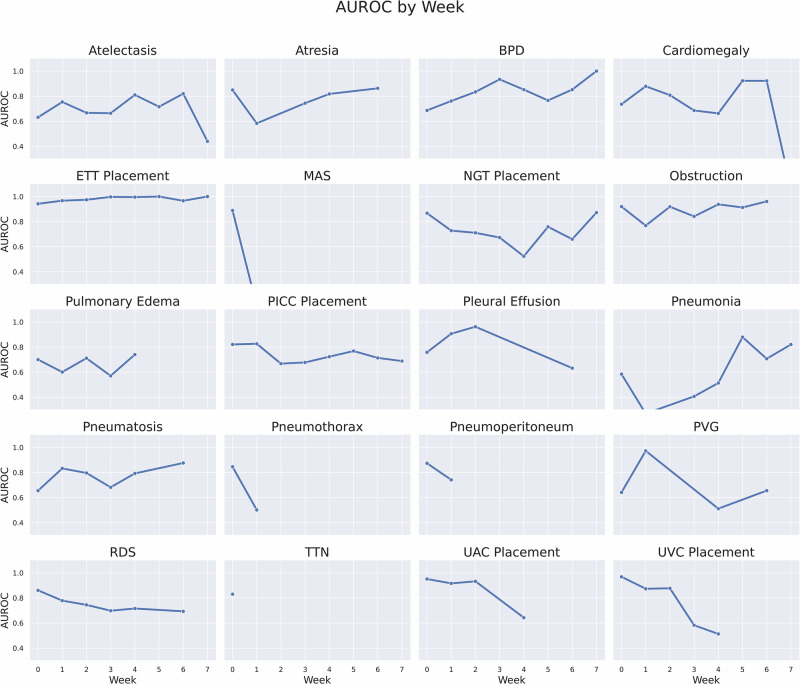
AUROC of NeoCLIP by the week image was obtained during admission. This figure illustrates the temporal performance stability of the NeoCLIP model, showing AUROC values across different postnatal weeks for each of the 20 radiological findings and device labels. Each line represents one label, allowing visualization of consistency or variability in model performance throughout a patient’s NICU stay. Most labels exhibit stable performance, but notable fluctuations include an increase in pneumonia detection accuracy after week 4 and a decline in umbilical vein catheter (UVC) detection performance after week 2, reflecting clinical changes in disease presentation and device usage over time.

**Fig. 2:**
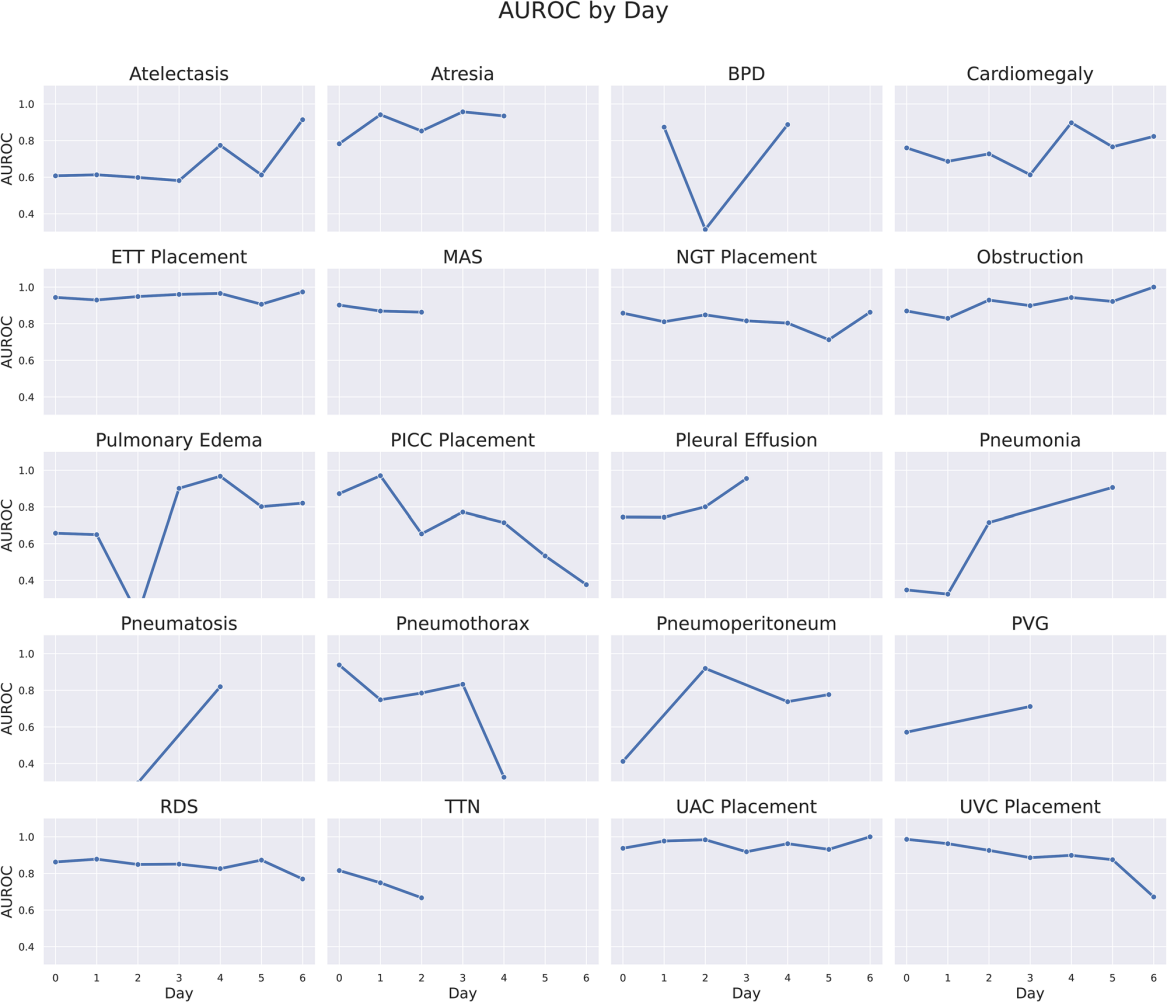
AUROC of NeoCLIP by the day image was obtained during first week of life. This figure details NeoCLIP’s diagnostic performance by postnatal day during the first week of life, stratified by each of the 20 labels. The goal is to assess how model performance varies during early neonatal life. While most AUROC trajectories remain stable, pneumothorax shows a marked decline after day 3, and pneumonia shows increased performance starting around day 4. These trends reflect clinical realities of disease timing and offer insight into model reliability during the most critical early days of neonatal care.

**Fig. 3:**
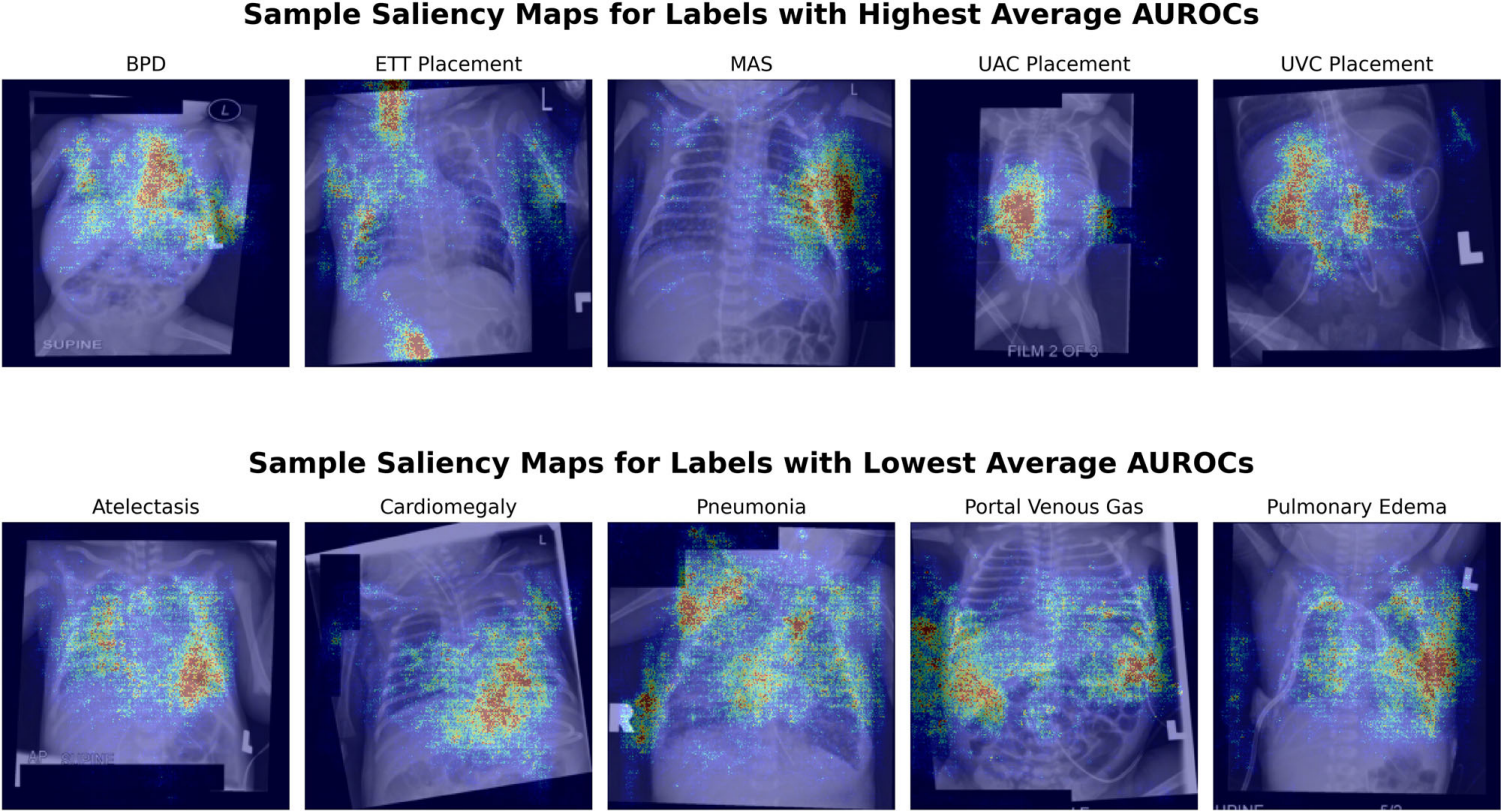
Sample saliency maps for the best and worst performing labels in NeoCLIP. Representative saliency maps are displayed for five of the best-performing labels and five of the worst-performing labels. The red-highlighted regions denote areas of highest attention used by the model to make predictions, while blue indicates minimal attention. High-performing labels show focused and anatomically appropriate attention, whereas low-performing labels demonstrate dispersed or ambiguous attention patterns, indicating possible model uncertainty or the diffuse nature of the pathology.

The performance of the large language model (LLM) extraction was assessed by two neonatologists. The kappa statistic measuring interrater reliability was 0.81 (95% CI, 0.72–0.91). In instances of disagreement, the reviewers resolved discrepancies through consensus. The overall accuracy of the LLM-extracted labels was 0.90, with perfect accuracy noted for seven labels. The sensitivity and specificity of the labels were 0.98 and 0.84, respectively. Table 2 summarizes the accuracy, sensitivity, and specificity for all 20 labels.For each of the twenty tasks, we determined the Area Under the Receiver Operating Characteristic (AUROC), which is summarized in Table 3. For the pathology labels, NeoCLIP performed best on BPD (0.94, SD = 0.01) and worst on pulmonary edema (0.69, SD = 0.04). The model performed better on medical device labels with its greatest AUROCs on ETT (0.97, SD = 0.01) and UVC placement (0.97, SD = 0.01). NeoCLIP outperforms both the logistic regression and ResNet-50 models on all labels. Additionally, NeoCLIP outperforms the BioViL model on all labels other than portal venous gas (0.73, SD = 0.07 v 0.77, SD = 0.06), but this difference is not statistically significant. The addition of gestational age and birthweight improved the performance of NeoCLIP in 11 of the 20 labels. However, this improvement was not statistically significant other than in cardiomegaly (0.68, SD = 0.02 v 0.73, SD = 0.02) and MAS (0.86, SD = 0.03 v 0.94, SD = 0.01).We also evaluated the consistency of NeoCLIP by analyzing AUROCs for all labels over time. Figure 1 summarizes the model’s performance by the week of the patient’s admission. AUROCs remained relatively stable other than pneumonia, which had an increase in AUROC after week 4, and UVC placement, which had a decrease in AUROC after week 2. We also evaluated the model’s performance for each day of the patient’s first week of admission (Fig. 2). Similar to the weekly analysis, not every label had images available for each day during the first week of life, but those that did showed a similar stability. The only major exception was pneumothorax which declined rapidly after day 3. We also generated saliency maps for the five best and worst performing labels as defined by AUROC (Fig. 3).

**Table 2:** Performance of large language model in label extraction

Label	Accuracy	Sensitivity	Specificity
Atelectasis	0.90	0.90	0.90
Intestinal Atresia	0.60	1.00	0.56
Bronchopulmonary Dysplasia	1.00	1.00	1.00
Cardiomegaly	0.95	1.00	0.91
Endotracheal Tube Placement	1.00	1.00	1.00
Meconium Aspiration Syndrome	0.85	1.00	0.77
Nasogastric Tube Placement	0.75	0.86	0.69
Intestinal Obstruction	0.65	1.00	0.59
Pulmonary Edema	1.00	1.00	1.00
Peripherally Inserted Central Catheter Placement	0.95	1.00	0.91
Pleural Effusion	0.90	0.9	0.9
Pneumonia	0.80	1.00	0.71
Pneumatosis Intestinalis	1.00	1.00	1.00
Pneumothorax	0.95	1.00	0.91
Pneumoperitoneum	0.85	1.00	0.77
Portal Venous Gas	1.00	1.00	1.00
Respiratory Distress Syndrome	0.85	1.00	0.77
Transient Tachypnea of the Newborn	1.00	1.00	1.00
Umbilical Artery Catheter Placement	0.95	1.00	0.91
Umbilical Vein Catheter Placement	1.00	1.00	1.00
**Total**	**0.90**	**0.98**	**0.84**

**Table 3:** Performance of NeoCLIP compared to multiple controls

Label	AUROC (SD)					
	NeoCLIP	NeoCLIP (with Gestational Age and Birthweight)	Logistic Regression	ResNet-50	BioVIL-T	BioVIL-T (with Gestational Age and Birthweight)
Atelectasis	0.70 (0.01)	0.71 (0.01)	0.55 (0.02)	0.69 (0.01)	0.69 (0.01)	0.69 (0.01)
Intestinal Atresia	0.88 (0.03)	0.92 (0.01)	0.54 (0.06)	0.75 (0.04)	0.76 (0.04)	0.75 (0.04)
Bronchopulmonary Dysplasia	0.94 (0.01)	0.95 (0.01)	0.85 (0.01)	0.92 (0.01)	0.93 (0.01)	0.94 (0.01)
Cardiomegaly	0.68 (0.02)	0.73 (0.02)	0.55 (0.03)	0.63 (0.02)	0.63 (0.02)	0.62 (0.02)
Endotracheal Tube Placement	0.97 (0.01)	0.97 (0.01)	0.64 (0.01)	0.83 (0.01)	0.84 (0.01)	0.84 (0.01)
Meconium Aspiration Syndrome	0.86 (0.03)	0.94 (0.01)	0.91 (0.02)	0.72 (0.03)	0.72 (0.03)	0.90 (0.02)
Nasogastric Tube Placement	0.88 (0.01)	0.88 (0.01)	0.69 (0.01)	0.74 (0.01)	0.74 (0.01)	0.75 (0.01)
Intestinal Obstruction	0.88 (0.02)	0.89 (0.02)	0.73 (0.02)	0.81 (0.03)	0.82 (0.02)	0.81 (0.02)
Peripherally Inserted Central Catheter Placement	0.85 (0.01)	0.86 (0.01)	0.66 (0.02)	0.70 (0.02)	0.72 (0.02)	0.73 (0.02)
Pleural Effusion	0.87 (0.02)	0.90 (0.02)	0.81 (0.02)	0.79 (0.02)	0.79 (0.02)	0.86 (0.02)
Pneumonia	0.75 (0.06)	0.75 (0.05)	0.50 (0.06)	0.67 (0.05)	0.66 (0.05)	0.63 (0.05)
Pneumatosis Intestinalis	0.89 (0.02)	0.89 (0.02)	0.55 (0.04)	0.88 (0.02)	0.88 (0.02)	0.86 (0.02)
Pneumothorax	0.93 (0.02)	0.93 (0.02)	0.81 (0.01)	0.82 (0.02)	0.83 (0.02)	0.86 (0.02)
Pneumoperitoneum	0.86 (0.03)	0.84 (0.04)	0.61 (0.01)	0.74 (0.05)	0.78 (0.04)	0.72 (0.06)
Portal Venous Gas	0.73 (0.07)	0.74 (0.08)	0.49 (0.12)	0.76 (0.06)	0.77 (0.06)	0.72 (0.09)
Pulmonary Edema	0.69 (0.04)	0.69 (0.04)	0.58 (0.04)	0.66 (0.04)	0.66 (0.04)	0.65 (0.04)
Respiratory Distress Syndrome	0.86 (0.01)	0.88 (0.01)	0.61 (0.01)	0.80 (0.01)	0.81 (0.01)	0.83 (0.01)
Transient Tachypnea of the Newborn	0.89 (0.02)	0.91 (0.01)	0.83 (0.02)	0.84 (0.03)	0.84 (0.03)	0.87 (0.02)
Umbilical Artery Catheter Placement	0.96 (0.01)	0.96 (0.01)	0.66 (0.02)	0.85 (0.01)	0.86 (0.01)	0.88 (0.01)
Umbilical Vein Catheter Placement	0.97 (0.01)	0.97 (0.01)	0.56 (0.02)	0.90 (0.01)	0.91 (0.01)	0.91 (0.01)

## Discussion

With this large cohort of infants, we developed NeoCLIP, the first self-supervised foundation model for the interpretation of neonatal radiographs. NeoCLIP successfully identified common neonatal pathologies and medical devices, performing comparably to similar models developed for adult and pediatric populations, despite being tasked with more distinct labels^15,16^. It outperformed logistic regression, BioViL, and ResNet-50 in all labels other than portal venous gas, although this difference was not statistically significant. While the addition of gestational age and birth weight did improve the performance of NeoCLIP, it was not statistically significant in the majority of labels.

NeoCLIP’s strong performance was expected given the model was pre-trained and fine-tuned on neonatal radiographs and reports, unlike ResNet-50 or BioViL. However, BioViL did outperform NeoCLIP in the identification of portal venous gas. While the exact reason for this superior performance is unclear, it may be attributed to the small number of positive cases for this condition (Table 1). Nevertheless, NeoCLIP’s overall advantage over ResNet-50 and BioViL demonstrates the importance of pre-training and fine-tuning a model for the population of interest. This is further reinforced by the fact that BioViL did not consistently outperform ResNet-50 in our cohort despite BioViL being pre-trained on adult radiographs whereas ResNet-50 is trained on a generalized database not limited to medical imaging. These findings underscore the importance of developing neonatal-specific models to ensure this vulnerable population receives the same level of care as adult and pediatric populations.

Our investigation reinforces the importance of gestational age and birth weight as key clinical risk factors for many neonatal pathologies^17–20^. While NeoCLIP significantly outperformed the logistic regression model based solely on these two factors, incorporating them into NeoCLIP further improved its performance (Table 3). While the improvement was not always statistically significant, it does demonstrate the importance of these clinical factors and highlight the potential value of a multimodal model, even for image-related tasks.

NeoCLIP performed better on medical device labels than those related to pathology. This was expected as the presence of these devices is a more distinct finding than many of the pathologic labels. This is reflected by the model’s lower performance on more subjective labels such as cardiomegaly and atelectasis when compared to well-defined radiographic findings such as pneumothorax or pneumatosis. However, the model was only tasked with detecting device presence, not assessing correct placement, a more complex and subjective task. Future work could explore NeoCLIP’s ability to evaluate the proper positioning of these critical lines and tubes. NeoCLIP’s performance remained relatively stable over time. When stratifying the images by week, we found the AUROCs remained consistent for the majority of labels (Fig. 1). However, more variability was noted when stratifying by day within the first week of life (Fig. 2). This discrepancy is likely a function of smaller sample size than inherent instability in the model as fewer images are captured per day than per week. While the overall stability is reassuring, it is important to examine labels where more inconsistency was noted, such as pneumonia and UVC placement. Pneumonia is typically diagnosed after the first few days of life, which is reflected by the model with its improved stability after day 3. Similarly, the AUROCs for pneumonia increase by weeks 5-7, indicating more infants are diagnosed during this period. Furthermore, UVCs are usually discontinued by day 10, which is consistent with the observed decline in AUROCs for UVC placement after this point. This time-series analysis provides valuable insight into how model performance may change as more or less data is available at different time points.To better explain the model’s performance, we generated saliency maps, where red highlights areas of highest attention and blue indicates lowest (Fig. 3). For the best performing labels, the model’s focus is highest on anatomically and physiologically relevant regions. For ETT and UAC placement, red areas align with the expected location of these devices on radiographs. For BPD and MAS, the model shows greatest focus on the lung fields. Additionally, in these best performing labels, the model demonstrates precision, with minimal scattered red foci. In contrast, the worst performing labels, though still accurate, are far less precise. In these cases, the model appropriately focused on anatomically relevant areas, such as the heart for cardiomegaly and liver for portal venous gas, but there were multiple red regions, suggesting ambiguity and reflecting the diffuse radiologic patterns typical of these conditions. While saliency maps cannot fully explain model behavior, they offer meaningful insights that support interpretability and may guide how such tools are deployed in clinical decision-making. The clinical utility of NeoCLIP lies in its potential to augment neonatal radiograph interpretation at the point of care, particularly in settings where radiologist input may be delayed or unavailable. In the NICU, timely interpretation of radiographs is critical as they are frequently obtained to confirm placement of life-sustaining devices or evaluate for emergent conditions. Based on its strong performance, NeoCLIP may be best positioned initially as a screening or confirmation tool for neonatologists, helping to validate line placement or flag urgent pathology while awaiting formal radiology interpretation. This may be especially valuable during overnight shifts, high-volume periods, or in resource-limited settings. While we do not envision NeoCLIP replacing radiologist interpretation, its use as a clinical decision support tool may reduce delays, improve patient safety, and provide an additional layer of assurance during clinical care. Ultimately, NeoCLIP could be integrated into a tiered interpretation workflow, confirming expected findings automatically and triaging uncertain or critical images for prioritized expert review. Before clinical adoption, however, additional studies are needed to validate performance across external and prospective cohorts.

Another key finding of our investigation was the novel use of a LLM to generate labels from radiograph reports. Since not all reports in our cohort contained disease-specific labels, we prompted a LLM to extract them from the text of the report. The observed accuracy, sensitivity, and specificity of the LLM in subgroup analysis demonstrate proof of concept for this methodology. To our knowledge, this approach has not been previously employed in the neonatal population and therefore represents a promising technique for labeling otherwise unlabeled images or data when developing deep learning models.Despite the strengths of this study, it is not without limitations. The model was developed and internally validated using single-center data. While this is a common methodological approach, it does limit the generalizability of the study. An important next step would be validating NeoCLIP on a cohort of infants from a geographically distinct NICU. Additionally, like all deep learning models, NeoCLIP faces inherent challenges with explainability. While saliency maps do provide some insight, they do not fully explain what image features drive identification. Another limitation of our study is our cohort’s heterogeneity in gestational age. We did not exclude any infants based on gestational age, resulting in a mean gestational age of 34 weeks at birth (Table 1). However, some of the pathology labels, such as RDS and BPD in preterm infants and TTN in term infants, are dependent on gestational age. A valuable next step would be to develop gestational age-specific models for these pathologies. However, there are some inherent challenges with this approach as any gestational age stratification would limit the size of the data pool for training. In our investigation, we already had small sample sizes for certain labels such as intestinal atresia, pneumoperitoneum, and portal venous gas without stratification (Table 1). While the concept of statistical power is not always consistent with deep learning studies, these lower sample sizes do present difficulties and challenge the validity of some of our results. The study also has inherent bias due to its retrospective design. Radiographs were obtained due to clinical indications, and therefore the very presence of a radiograph reflects provider concern for a disease process. However, this bias could only be mitigated by imaging all infants admitted to a NICU, which is not ethical nor feasible due to the potential harm of radiation. Despite these limitations, this investigation remains of the largest of its kind in neonates. Its novel methodology and promising results represent a major step in the application of deep learning principles to this vulnerable population.In this large retrospective study, we developed and validated NeoCLIP, a deep learning model that accurately identified a range of radiological findings relevant to neonatal intensive care. Performing comparably to models developed for adult and pediatric populations, NeoCLIP represents the first application of advanced deep learning methodologies to interpret neonatal radiographs. Our study is also the first to use a LLM to extract disease-specific labels from neonatal radiology reports to guide model fine-tuning. While NeoCLIP has limitations with generalizability and interpretability, it represents a major step in the application of AI to interpret medical data and improve neonatal care.

## Methods

### Study population

We conducted a retrospective study on a cohort of all infants admitted to the neonatal intensive care unit (NICU) at Beth Israel Deaconess Medical Center (BIDMC) in Boston, MA from January 2008 to December 2023. Any infant who did not obtain a radiograph during their admission to the NICU was excluded. For all the infants in our cohort, we collected radiographs and corresponding reports from their admission to the NICU, along with a predefined list of demographic data (Table 1). We used simple random sampling to then split the cohort into three distinct sets: training, validation, and test. The sampling was performed at the patient level to ensure that all radiographs and reports from a single patient remained in the same set. We used Python’s random module with a random seed of 1 for reproducibility. The requirement for informed consent was waived by the institutional review board at BIDMC. The study was performed in accordance with the principles of the Declaration of Helsinki.

### Model development

**Fig. 4:**
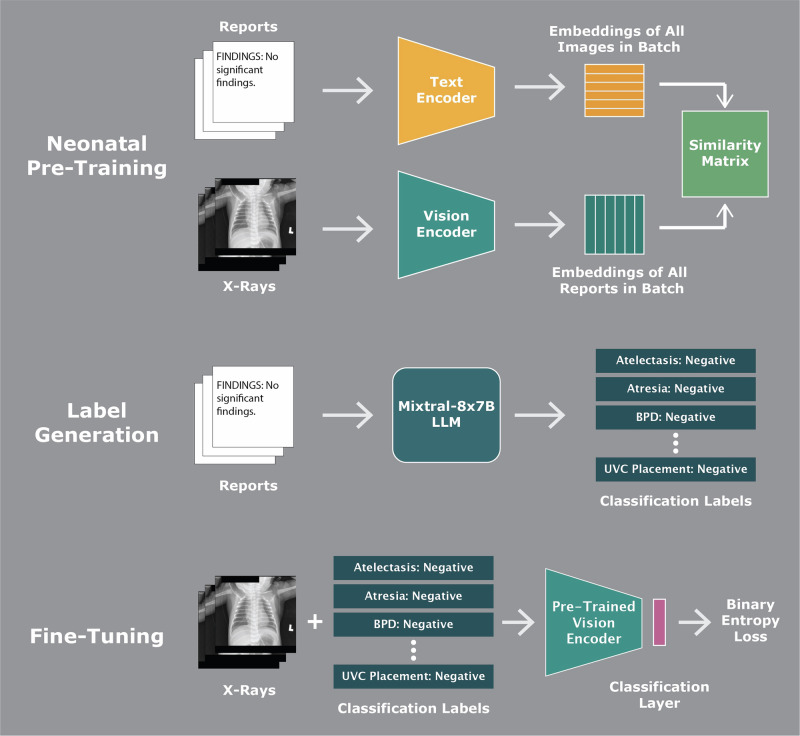
Steps of NeoCLIP model pre-training and fine-tuning. This schematic outlines the step-by-step development of the NeoCLIP model. Initially, neonatal radiographs and their associated reports are used in a self-supervised contrastive pre-training phase, aligning image and text embeddings in a shared latent space using cosine similarity. Next, the model undergoes supervised fine-tuning using structured labels extracted from reports via a large language model, aided by few-shot prompting. A multilayer perceptron and classification head are appended to the vision encoder to enable binary classification across 20 distinct labels. An optional multimodal enhancement includes gestational age and birth weight concatenated to the image features for improved predictive accuracy.

We developed NeoCLIP, a deep learning multitask model capable of identifying the presence of 15 neonatal radiological signs/features pathologies and 5 medical devices on neonatal radiographs. The findings pathologies included intestinal atresia, BPD, cardiomegaly, meconium aspiration syndrome (MAS), pneumonia, RDS, transient tachypnea of the newborn (TTN), atelectasis, intestinal obstruction, pulmonary edema, pleural effusion, pneumatosis intestinalis, pneumothorax, pneumoperitoneum, and portal venous gas. The medical devices include endotracheal tube (ETT), nasogastric tube (NGT), peripherally inserted central catheter (PICC), umbilical artery catheter (UAC), and umbilical vein catheter (UVC).To build NeoCLIP, we adapted BioViL, a domain-specific vision-language model trained on adult chest X-rays and their corresponding reports^21^. BioViL uses a fine-tuned version of ResNet-50 as its vision encoder and BERT, a LLM, as its text encoder^22^. ResNet-50 is a deep learning model pre-trained on ImageNet, a large-scale imaging dataset containing millions of diverse images not specific to medicine^23^. We modified this architecture by pre-training NeoCLIP on our dataset of neonatal radiographs and corresponding reports, as shown in Fig. 4. Image and text embeddings generated by the encoders were projected to a shared latent space, and a cosine similarity score:

$${s}_{i,j}=\frac{{{v}_{i}}^{T}\cdot {t}_{j}}{|{v}_{i}|\,|{t}_{j}|}$$

### Cosine similarity score

was calculated for a given pair of image i and report j. Using standard contrastive language-image pre-training training approaches, the models were trained to maximize the similarity scores of the true image-text pairs while minimizing the scores of other pairs^24,25^. Training was conducted for 50 epochs with a batch size of 256, using the Adam optimizer with an initial learning rate of 0.0001. To mitigate overfitting, we applied data augmentation techniques such as random rotation, horizontal and vertical flips, affine transformations, and color jittering^26,27^. Additionally, to enhance training efficiency, all images were resized to 224×224 pixels. The pixel values were normalized using a mean of [0.485, 0.456, 0.406] and a standard deviation of [0.229, 0.224, 0.225], following the standard ImageNet preprocessing convention for pretrained ResNet models^28–30^.

After pre-training, we then fine-tuned NeoCLIP using supervised training techniques. Since disease-specific labels were not readily available in the dataset, we employed Mixtral-8x7B, a LLM, to extract these labels from the radiology reports using structured prompts developed from descriptions provided by two neonatologists (Table 4). To further improve performance, we used “few-shot prompting,” including a small set of positive and negative training examples for each label within the prompt. After the labels were generated, we used stratified sampling to select 10 positive and 10 negative labels for each finding, which were independently reviewed by our neonatologists for accuracy, specificity, and sensitivity.We then continued the task-specific fine-tuning of NeoCLIP using the LLM-generated labels as targets. The vision encoder was modified by adding a multilayer perceptron and a final linear layer as a classification head. Additionally, we fine-tuned a version of NeoCLIP where the gestational age and birth weight of patients, standardized to zero mean and unit variance, were concatenated to the vision encoder’s output.

**Table 4:** Prompt template for large language model label extraction

**Prompt Template**	Read the following neonatal CXR report and determine whether the presence of the following finding was explicitly mentioned: **<FINDING>**. **<Example language>**. Output just a ‘yes’ or ‘no’. **<FEW-SHOT EXAMPLES>** **<TEST _EXAMPLE>** Answer:
**Few-Shot Template**	Examples [random order]: **<Pos. Example 1>** Answer: yes **<Pos. Example 2>** Answer: yes **<Neg. Example 1>** Answer: no **<Neg. Example 2>** Answer: no
**Full Prompt Example**	Read the following neonatal CXR report and determine whether the presence of the following finding was explicitly mentioned: Atelectasis. For example, “chest X-ray reveals segmental or lobar collapse”. Output just a ‘yes’ or ‘no’. **<FEW-SHOT EXAMPLES**> <**TEST _EXAMPLE>** Answer:

After fine-tuning, we then trained NeoCLIP with a binary cross-entropy loss function to optimize the detection of the radiological specific neonatal findings. The training was conducted for 50 epochs, with a batch size of 256, using the Adam optimizer and a learning rate of 0.00005. The final model checkpoint was selected based on the highest average AUROC curve on the validation set.

### Model benchmarks

We evaluated the performance of NeoCLIP against several standard baseline models, including a logistic regression model based solely on the gestational age and birth weight, the BioViL model, and the ResNet-50 model. Both the BioViL and the ResNet-50 model were fine-tuned using the same training data, loss function, and hyper-parameters as the contrastive learning model. To assess the consistency of NeoCLIP, we examined whether its performance varied based on the infant’s chronologic age at time of radiograph. We also generated saliency maps for the best and worst performing labels to better understand the radiographic features influencing the model’s prediction. These maps highlight key regions of an input image that drive the model’s decision-making.

## Data Availability

Due to the sensitive nature of pediatric health data and additional privacy protections for minors, individual participant data are not available for sharing. Summary-level data supporting the conclusions of this article may be available upon reasonable request and with appropriate institutional and ethical approvals.

## Code availability

Custom analysis scripts are available from the corresponding author upon request. Standard statistical analyses were performed using Python version 3.10 with publicly available packages listed in the Methods section.
